# Supplementary material for: Context-Dependent Role of Glucocorticoid Receptor Alpha and Beta in Breast Cancer Cell Behaviour
Source: Cells. 2023 Mar 1;12(5):784. doi: 10.3390/cells12050784 (PMC10000936; doi:10.3390/cells12050784)
Supplement: Supplementary file 1 [file cells-12-00784-s001.zip › cells-2163356-supplementary.pdf]

Supplementary Table S1. Histologic characteristic of samples used for GRtotal and GRβ immunohistochemistry

Tumor histology	Nottingham histologic grade	TIL (%)	TN	Notthingham prognostic index	ER	PR	Her2	Ki67 (%)
Invasive carcinoma NST	grade II	5	pT1c N0	3.28	pos	pos	neg	2
Invasive carcinoma NST	grade I	5	pT2 N1	3.42	pos	pos	neg	3
Invasive mucinosus carcinoma	grade II	0	pT2 N0	3.48	pos	pos	neg	3
Invasive carcinoma NST	grade II	5	pT1c N0	3.36	pos	pos	neg	5
Invasive carcinoma NST	grade III	10	pT2 N1a	5.96	pos	pos	neg	25
Invasive carcinoma NST	grade I	0	pT1c N0	2.26	pos	pos	neg	5
Inasive lobular carcinoma	grade II	5	pT3 N1a	5.3	pos	pos	neg	3
Invasive carcinoma NST	grade II	0	pT1c N0	3.3	pos	pos	neg	3
Invasive carcinoma NST	grade II	5	pT1c N0	3.26	pos	pos	neg	20
Inasive lobular carcinoma	grade II	5	pT2 N1	4.72	pos	pos	neg	2
Invasive carcinoma NST	grade III	na	cT1c cN0		pos	pos	neg	15
Invasive carcinoma NST	grade III	15	pT1c N0	4.28	neg	neg	neg	75
Invasive carcinoma NST	grade II	5	pT1c N0	3.26	neg	neg	neg	15
Invasive carcinoma NST	grade III	10	ypT2 N0	4.9	neg	neg	neg	30
Invasive carcinoma NST	grade III	10	pT1c N0	4.32	neg	neg	neg	30
Invasive carcinoma NST	grade III	40	pT1c N0	4.38	neg	pos	neg	85
Invasive carcinoma NST	grade III	20	pT1c N0	4.3	neg	neg	neg	50
Invasive carcinoma NST	grade III	5	pT1c N0	4.24	neg	neg	neg	30
Invasive apocrine carcinoma	grade II	5	pT2 N1	4.44	neg	neg	neg	10
Invasive carcinoma NST	grade III	5	pT3 N1	6.56	neg	neg	neg	60

NST: no special type

Nottingham histologic grade: Grade I is assigned for a total score of 3 to 5 (well differentiated). Grade II is assigned for a total score of 6 to 7 (moderately differentiated). Grade III is assigned for a total score of 8-9 (poorly differentiated).

TIL: tumor infiltrating lymphocyte

T: tumor size; N: spread of cancer to nearby lymph nodes

Notthingham prognostic index: I (excellent) ≤2.4; II (good) >2.4 but ≤3.4; III (moderate) >3.4 but ≤5.4; and IV (poor) >5.4.

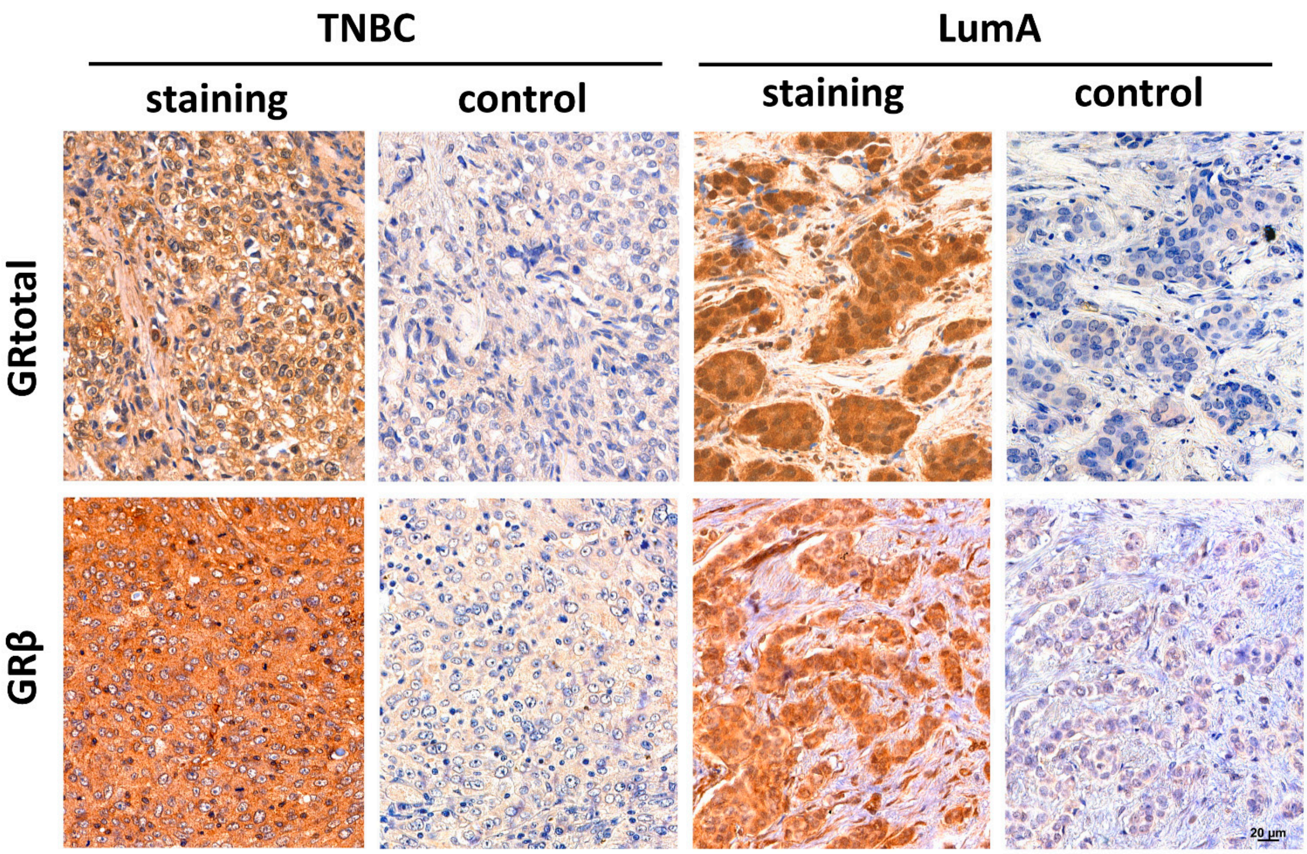

Supplementary Figure S1. Glucocorticoid receptor staining specificity using N-terminal specific antibody reflecting and referred as (GRtotal) and a selective antibody specific against the GRβ isoform (GRβ), indicated as staining in triple-negative and luminal A type breast cancer. Controls are presented as negative control omitting the primary antibody.

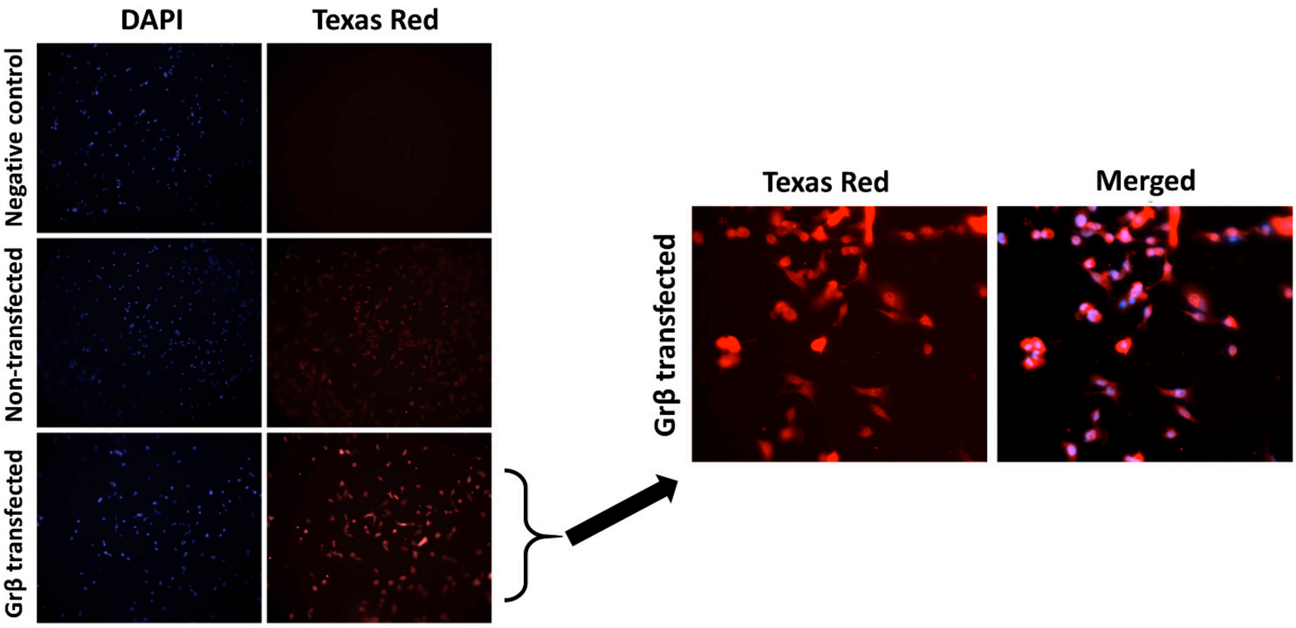

Supplementary Figure S2. Immunocytochemistry using anti-GRβ antibody. GRβ is mainly localized in the cytoplasm following transfection
